# Models of Perinatal Palliative Care for Pregnant Women and Their Fetuses with Life-Limiting Conditions: A Literature Review

**DOI:** 10.3390/jpm16070353

**Published:** 2026-06-30

**Authors:** Daniela Valle Almeida Figueredo, Silvia de Lourdes Loreto Faquini, Edward Araujo Júnior, Tammy Caram Sabatine, Gustavo Yano Callado, Antonio Braga, Roberta Granese, Alex Sandro Rolland Souza

**Affiliations:** 1 Service of Fetal Medicine, Institute of Integral Medicine Prof. Fernando Figueira (IMIP), Recife 50070-555, PE, Brazil; danivallealmeida@gmail.com (D.V.A.F.); sfaquini@yahoo.com.br (S.d.L.L.F.); alexrolland@uol.com.br (A.S.R.S.); 2 Discipline of Woman Health, Municipal University de São Caetano do Sul (USCS), São Caetano do Sul 09521-160, SP, Brazil; araujojred@terra.com.br (E.A.J.); tammy.caram@uscsonline.com.br (T.C.S.); 3 Department of Obstetrics, Paulista School of Medicine-Federal University of São Paulo (EPM-UNIFESP), São Paulo 04023-062, SP, Brazil; 4 Faculdade Israelita de Ciências da Saúde Albert Einstein, Hospital Israelita Albert Einstein, São Paulo 05652-900, SP, Brazil; gycallado@gmail.com; 5 Department of Gynecology and Obstetrics, School of Medicine, Federal University of Rio de Janeiro (UFRJ), Rio de Janeiro 22240-003, RJ, Brazil; bragamed@yahoo.com.br; 6 Department of General and Specialized Surgery, School of Medicine and Surgery, Federal University of the State of Rio de Janeiro (UNIRIO), Rio de Janeiro 22290-240, RJ, Brazil; 7 Postgraduate Program in Applied Health Sciences, University of Vassouras (Univassouras), Vassouras 27700-000, RJ, Brazil; 8 Department of Biomedical and Dental Sciences and Morphofunctional Imaging, “G. Martino” University Hospital, 98100 Messina, Italy

**Keywords:** perinatal palliative care, fetal malformations, prenatal diagnosis, life-limiting fetal conditions

## Abstract

**Objective**: To review the literature on palliative care protocols and models of care for pregnant women and their fetuses with life-limiting conditions. **Methods**: A narrative literature review was conducted in the PubMed/MEDLINE and Virtual Health Library (VHL)—BIREME/SciELO/LILACS, using the descriptors “palliative care” and “prenatal care”. Studies of all designs published between February 2015 and May 2025 were considered for inclusion. Articles in languages other than Portuguese, English, and Spanish, duplicates, and those that did not discuss care protocols or experiences in perinatal palliative care for life-limiting fetal conditions starting from prenatal care were excluded. Articles were selected through title, abstract, and full-text screening. **Results**: Twenty-one studies focused on prenatal care were selected, presenting protocols and experiences of care in palliative fetal medicine. Most addressed the diagnosis of life-limiting fetal malformations, prenatal care, birth and delivery plan, perinatal grief and the puerperium. Across the included studies, a recurring emphasis on individualized, patient and family-centered approaches was identified, reflecting core principles of personalized medicine. Tailoring communication, care planning, and bereavement support to the specific clinical, genetic, cultural, and psychosocial profile of each dyad emerged as a structural characteristic of effective perinatal palliative care models. **Conclusions**: There is a scarcity of specific palliative care protocols for pregnancy, indicating a need to expand studies. The reviewed literature can contribute to the creation and adaptation of palliative care protocols and models for pregnant women and their fetuses with life-limiting conditions, may support more consistent care planning, improved communication, and better alignment with parental values.

## 1. Introduction

Palliative care is most associated with the adult population. According to the World Health Organization (WHO), in a concept created in 1990 and updated in 2002, “palliative care consists of assistance promoted by a multidisciplinary team, aiming at improving the quality of life of the patient and their families facing a life-threatening illness, through the prevention and relief of suffering, early identification, impeccable assessment and treatment of pain and other physical, social, psychological, and spiritual symptoms” [1].

In 1982, a paper was published in the *American Journal of Diseases of Children* with the objective of implementing palliative care (PC) in neonatal intensive care units (NICUs) for newborns with poor prognosis [2]. Subsequently, the concept of perinatal palliative care began to be applied in medicine in the 1990s [3]. In 1997, the idea of perinatal hospices or perinatal palliative care emerged with the aim of expanding treatment options for newborns with a prenatal diagnosis of a life-limiting condition, instead of only proposing termination of pregnancy [4].

Only in 2001 was this concept further extended to prenatal care, with a care model published in the American Journal of Obstetrics and Gynecology for families who wished to continue a pregnancy following a diagnosis of a lethal fetal malformation. The goal was to place the family as the central point of care, rather than the malformation itself, giving the family the opportunity to experience the birth and the process of death with the minimum possible interference [5].

In Brazil, until 2017, there were no widely disseminated or standardized protocols for integrating palliative care into prenatal care. One of the first structured models was developed at the University of São Paulo, which includes as candidates any family with a diagnosis of a malformation that endangers fetal life. This model was not the only initiative in the country, but it represented an important step toward systematizing care. It introduced key elements such as early referral to palliative care at the time of diagnosis, multidisciplinary follow-up, structured birth planning, and integration of psychological and grief support [6]. These components contributed to changes in clinical practice by promoting family-centered care, improving communication, and aligning medical conduct with parental values and preferences.

Thus, the objective of this article was to conduct a literature review on palliative care protocols in fetal medicine for pregnant women and their fetuses with life-limiting conditions. The justification for conducting a review on this topic lies in the scarcity of protocols in institutions and the limited training of health professionals in this field [7,8]. A synthesis of the available evidence can support the development and adaptation of protocols, which are required by Brazilian health policy (resolution CFM 1.931/2009), promoting the qualification of perinatal care in situations involving life-limiting conditions.

In this review, the term ‘perinatal palliative care’ is used as the standard terminology to describe a comprehensive approach that spans from prenatal diagnosis through post-natal care and bereavement support. Related terms such as ‘fetal palliative care’ and ‘perinatal hospice’ are used in specific contexts when referring to particular models or settings described in the literature.

For the purposes of this review, ‘life-limiting condition’ refers to any fetal condition associated with a very high probability of death in utero, at birth, or in early neonatal life, or with survival only under conditions of severe and irreversible morbidity. The term ‘life-threatening condition’ may imply potential for cure or long-term survival with treatment, while ‘lethal condition’ is reserved for diagnoses with near-certain antenatal or early neonatal death. These distinctions are not uniformly applied across the included literature, and terminological variability is acknowledged as a limitation of this field.

## 2. Methods

A narrative literature review was conducted to synthesize current evidence on perinatal palliative care, with a focus on care models for pregnant women carrying fetuses diagnosed with life-limiting conditions. This narrative design was chosen due to the heterogeneity of the available literature, which includes diverse study designs (qualitative studies, case reports, institutional protocols, and expert recommendations), as well as variability in definitions and outcomes. This diversity limits the feasibility of conducting a formal systematic review with quantitative synthesis.

The literature search was performed in two electronic databases, PubMed/MEDLINE and Virtual Health Library (VHL) (BIREME/SciELO/LILACS), using the following descriptors: “prenatal care” AND “palliative care”. The descriptors could be present in the title, abstract, or subject. Citation tracking (snowball sampling) was also used, in which citations in the articles were included if they appeared relevant to the review. Study selection and screening were conducted by a single reviewer. Titles were first screened for relevance and language, followed by abstract screening, and then full-text reading for eligibility. A structured data extraction table was used to organize key information from included studies (authors, country, year, main findings). No formal data extraction form was used, and no independent inter-rater reliability assessment was performed, which is consistent with the narrative design but represents a methodological limitation. This structured narrative review was guided by the SANRA (Scale for the Assessment of Narrative Review Articles) framework. Specifically, the review aimed to meet SANRA criteria related to: (1) clearly defined objective; (2) appropriate literature selection with explicit inclusion and exclusion criteria; and (3) structured synthesis of evidence. However, no formal domain-by-domain SANRA self-assessment was performed, which represents a limitation in methodological transparency.

All study designs from February 2015 to May 2025 involving the selected descriptors were considered for inclusion. Inclusion criteria comprised studies addressing perinatal palliative care during the gestational period, with suggested models or guidelines for care in cases of fetal diagnosis of life-limiting conditions. Studies that did not discuss perinatal palliative care during the prenatal care period, did not present guidelines for creating care models for pregnant women with a fetal diagnosis of a life-limiting condition, that were written in languages other than English, Portuguese, and Spanish, or that were exclusively about palliative care related to a single specific disease without broader applicability and range, were excluded.

For topics beyond perinatal palliative care, such as concepts, definitions, communication of bad news, management of high-risk prenatal care, and legislation, complementary bibliography was used. The complementary material was consulted in the same databases used, through the snowball technique, and from books and the literature considered relevant to the topic.

The search pages were saved in .pdf format for title and relevance screening and were subsequently imported into a table in Microsoft^®^ Word (Version 2025) to support data extraction and narrative synthesis.

## 3. Results

**Figure 1:**
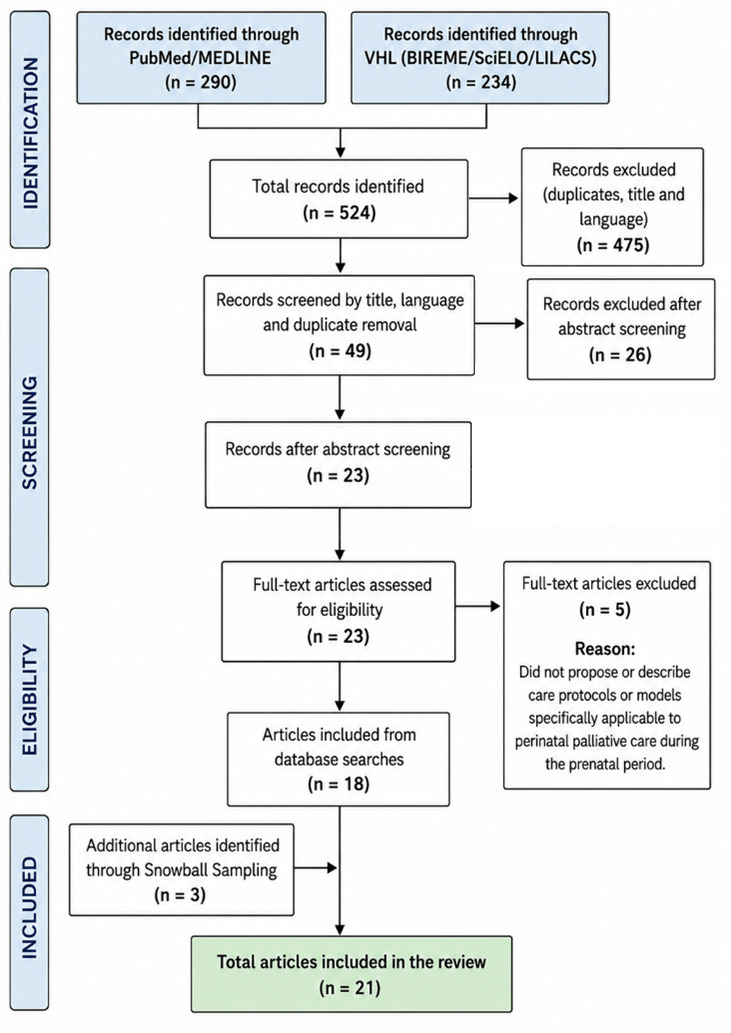
Flowchart of article selection in PubMed/MEDLINE and Virtual Health Library (VHL).

A total of 290 records were identified in PubMed/MEDLINE and 234 in VHL (BIREME/SciELO/LILACS). Following title and language screening, 49 records were selected for abstract reading, 47 of them duplicated. After abstract screening, 23 records were deemed eligible for full-text assessment, 22 of them duplicated in both databases and one was identified only in the VHL. Five articles were excluded at this stage, all of which were duplicates in both databases. Thus, 18 articles with models and guidelines for palliative care in prenatal care, for cases of life-limiting fetal conditions, were selected (17 were duplicates in both databases). Three articles were also added to the review using the snowball technique, resulting in a total of 21 articles to be used as a basis for the review’s elaboration (Figure 1).

**Figure 2:**
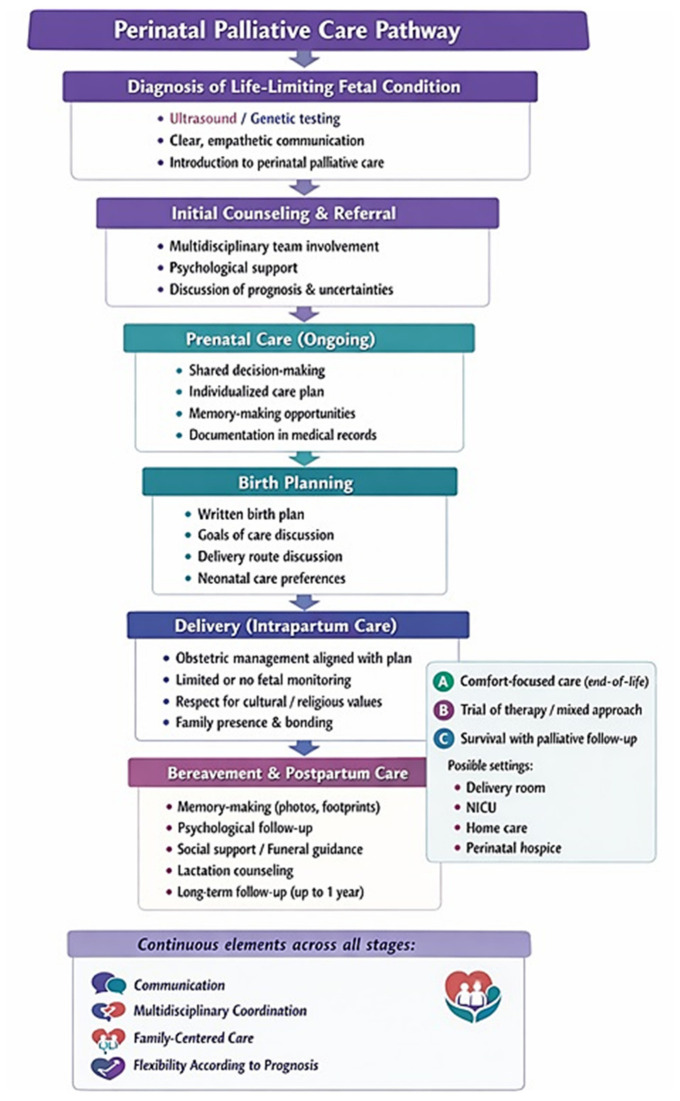
Conceptual synthesis of key components identified across published models of perinatal palliative care.

In addition, a proposal path for perinatal care from prenatal to postpartum was created. Presents a conceptual synthesis derived from recurring elements identified across the reviewed studies and should not be interpreted as a validated clinical pathway (Figure 2).

For data systematization, the authors, country, and content of the article were listed, identifying information or guidelines for perinatal palliative care models for the prenatal period (Table 1).

The present review considered some characteristics of the published studies (Table 1). Most of the articles selected were from USA (n = 11) [4,6,8,9,11,12,14,15,18,19,23], followed by four from Brazil [3,7,17,22], two from Italy [16], two from Poland [21,24], one from United Kingdom [10] and one from Spain. Of these, ten studies provide lists or charts with the malformations eligible for palliative care, including life-limiting fetal or neonatal conditions [7,10,13,14,17,18,19,20,21,24].

Regarding publication year, studies were distributed as follows: two from 2016 [9,10], two from 2017 [7,11], one from 2018 [3], four from 2019 [6,12,13,14], six from 2020 [4,15,16,17,18,19], one from 2022 [20], two from 2023 [21,22], one from 2024 [23], and one from 2025 [24]. This distribution reflects a growing interest in perinatal palliative care, particularly from 2019 onwards.

Of the included studies, five are literature reviews [7,13,16,19,22]; two contain recommendations from specialist societies, the American College of Obstetricians and Gynecologists (ACOG) and the Sociedad Española de Neonatología (SENEO) [20]; five provide recommendations for palliative care starting from prenatal care, according to the authors’ experience [8,10,12,15,23]; three relate to a model of care adopted in a specific health institution [3,11,18]; two derived from chart reviews [17,24]; and four resulting from interviews and questionnaires with the participation of family members [4,9,14,21], one of which also included the opinion of specialists [14].

To provide an organized reference, due to the difficulty in defining a life-limiting fetal condition, the definitions found in the review were highlighted. Of the selected studies, ten provide an explicit conceptual definition of “life-limiting fetal condition” (Table 2) [3,4,6,9,10,16,18,19,20,21]. It is noteworthy that the other studies did not make the definition of a life-limiting condition clear, describing it in a narrative form, or providing excerpts that address severe diseases, risk of death, or implicitly suggesting candidates for perinatal palliative care. The included studies are methodologically heterogeneous, comprising narrative reviews, expert recommendations, qualitative studies, and institutional protocols. This diversity reflects the developmental stage of the field but limits direct comparability between studies. As a result, the synthesis prioritizes conceptual convergence rather than methodological hierarchy.

Analyzing the process experienced by the families of fetuses affected by conditions considered life-limiting, the present review provides a line of care, from the moment of diagnosis during the gestational period until birth and grief attention, based on the works found in the researched literature.

Perinatal palliative care is considered an emerging concept [6,7,10,13,17]. Candidates are patients with fetal or neonatal life-limiting conditions, and the focus is on the quality of life of the newborn and their families, regardless of their time of life [3,4,6,7,13,17,18,20]. It is widely recommended that patients be referred for palliative care at the time of diagnosis [3,10,16,17,24]. The consultation provides the opportunity to welcome the family and begin the elaboration of anticipatory grief [3,9,11,14].

### 3.1. Diagnosis of a Fetal Life-Limiting Condition

The diagnosis of a fetal life-limiting condition will be made through ultrasonography, preferably by a fetal medicine specialist, or by complementary exams such as genetic tests, that include invasive (chorionic villus sampling, amniocentesis and cordocentesis, to perform the fetal karyotype or microarray), according to availability; or through non-invasive screening tests (non-invasive prenatal test (NIPT) [4,6,10,19,22]. The moment of diagnosis is of great importance because it generates anxiety and apprehension in families [3,4,10,11,17]. The loss of the idealized child or of the future plans causes the grief to begin at this moment [3,9,11,15,19,22]. Communication should be individualized, clear, and sincere, and excessive technical terms should be avoided [10,15,20]. The majority of the studies found in the review recommend training in palliative care and communication for perinatal healthcare professionals [4,6,7,12,13,14,16,18,20,22,23,24].

From a personalized medicine perspective, the diagnostic phase is particularly significant: the precise characterization of the underlying condition, whether structural, chromosomal, or monogenic, has direct implications for prognosis counseling, recurrence risk estimation, and individualization of the care plan. Advances in genomic prenatal diagnostics, including chromosomal microarray analysis and, increasingly, exome sequencing, enable a more precise molecular characterization of fetal anomalies, thereby informing more nuanced and individualized prognostic communication. This genetic precision not only allows families to receive more accurate information about the expected clinical course but also facilitates the identification of conditions with potential therapeutic implications or research relevance, which may inform parental decision making within the palliative care framework.

It is recommended that, during the conversation, professionals explain the fetal malformation, its current state, and how it is likely to evolve, answering the questions of the parents and family members. They should also provide guidance on the planned follow-up and explain what palliative care is, informing which conducts will be adopted [3,6,7,9,10,15,18,19,20,24].

It is worth noting that the importance of structured perinatal care pathways extends beyond the identification of major structural anomalies. First-trimester prenatal screening may also identify pregnancies at increased risk of adverse neonatal outcomes, reinforcing the value of early referral and coordinated care planning [25]. In the context of perinatal palliative care, the ultrasound and genetic triage of the first trimester allow for an early palliative care plan when severe or limiting conditions are identified already in this period, such as anencephaly, bilateral renal agenesis or trisomy 18 or 13, for example.

### 3.2. Prenatal Care

A multidisciplinary team is the foundation of palliative care. With the prenatal diagnosis of a life-limiting condition, counseling should be led by a range of professionals, which may include an obstetrician, fetologist, neonatologist, palliative pediatrician, psychologist, social worker, anesthesiologist, surgeon, religious representative, among others, depending on the identified condition [3,4,6,7,10,11,12,13,16,18,19,20,21,23,24]. The family should be informed about palliative care and, together with the team, build a care plan that must be followed, regardless of the professionals present at the time of delivery [3,6,16,17]. It is of great value to write all consultations and conversations in the medical chart, facilitating communication among professionals [3,4,10,13,14,19,23,24].

It is essential to welcome and acknowledge the feelings of the pregnant woman and her family, reinforcing their role as caregivers [3,11]. The parenting experience exists even in the face of an adverse outcome, making it important to value their choices, attribute meaning to the pregnancy, and record moments that become memories [11,19,21,23]. Imaging exams and prenatal follow-up can contribute to this, such as through three-dimensional (3D) ultrasonography, audio and video recording of fetal heartbeats, and photographic registration [4,11,18,19,20].

### 3.3. Birth Plan

All authors recommend writing a birth plan. This promotes a greater sense of control for caregivers [7,11,12,14,16,18,19,21]. In perinatal palliative care, this plan goes beyond birth, also including prenatal care and follow-up after perinatal loss, if applicable [6,7,8,9,11,15,16,19]. It must be built jointly by the family and the healthcare team [6,7,19,22,24]. It must contain the baby’s name, fetal diagnosis, route of delivery, neonatal care, and the family’s preferences [7,11,14,17,18,19,20].

### 3.4. Delivery

Vaginal delivery is recommended because it is a physiological process with lower maternal risk [7,13,21]. However, cesarean section may be considered in the presence of obstetric indications or an informed maternal request, after careful discussion of risks and benefits [6,7,12,18,19].

Intrapartum management should be aligned with the previously established birth plan. Fetal monitoring may be reduced or omitted, depending on the goals of care. Respect for cultural and religious values is essential at this stage [3,4,6,7,8,14,19,20,23]. The presence of family members, privacy, and opportunities for bonding should be ensured, including immediate contact with the newborn whenever possible [3,6,7,11,14,18,22].

### 3.5. Birth

The approach to neonatal care should be defined in the birth plan and reassessed after birth according to diagnostic confirmation and the newborn’s clinical condition [3,4,6,7,9,10,11,12,14,18,19,20,24]. In cases where exclusive palliative care is indicated, comfort measures (pharmacological or non-pharmacological) should be adopted [4,6,7,12,13,18].

Depending on clinical evolution, different care pathways may be followed, including end of life care in the delivery room, admission to a neonatal unit, discharge with home-based palliative care, or referral to a perinatal hospice where available [6,7,9,10,12,16,18,20,24]. Some conditions may present longer than expected survival despite significant morbidity, reinforcing the need for ongoing reassessment and individualized care planning [9,10,12,16].

### 3.6. Perinatal Grief and Puerperium

In case of perinatal death, healthcare teams can offer moments to create memories, such as: taking photos, getting a lock of hair, dressing the baby, giving a bath, making hand or footprint stamps, and providing hospital information leaflets, all according to the wishes of the woman giving birth and the availability of professionals at the time of delivery [3,6,9,11,12,16,20]. Referral to Psychology is essential, given that it is necessary to follow-up with families in order to detect complicated grief and offer appropriate treatment [9,11,13,20].

It is necessary to provide information about the bureaucratic issues concerning the transportation and burial of the baby’s body [7,9,13,14,16,19]. In some cases, evaluate autopsy [7,14,16,19]. Organ donation is also a theme highlighted in the literature [10,16,18,19]. The social service can be called upon and be present in the care, investigating the need for funeral assistance and to help organize the family support network [11,20].

The grief process can be made difficult by the physiological changes in the body during the puerperium. If the patient requests it, lactation suppression can be performed. However, milk donation is an option for those who wish to do so [9,20,22]. Follow-up is recommended in the postpartum period, which can be done through a call, letter, or holding events for bereaved families, among other actions [3,4,7,12,13,17,18,23]. Some authors recommend follow-up up to one year after perinatal loss [3,7].

## 4. Discussion

The use of a flexible, prognosis-based definition of life-limiting conditions, as proposed by ACOG [6], may better reflect the clinical reality of fetal medicine, encompassing lethal fetal conditions as well as others for which there is little or no prospect of long-term extrauterine survival without severe morbidity or extremely low quality of life and for which there is no cure. This approach encompasses a range of conditions associated with a high risk of death or survival with severe morbidity, without relying on rigid or time-based criteria. By acknowledging the inherent uncertainty of prognostic assessment in the perinatal setting, it supports more individualized, ethically grounded, and patient-centered care planning, which is central to the integration of perinatal palliative care.

A commonly cited framework is the classification proposed by Leuthner, in 2004, which offers a conceptual framework for decision making in perinatal palliative care based on diagnostic and prognostic certainty [26]. It describes three groups: conditions with a certain diagnosis and prognosis; conditions with an uncertain diagnosis but a likely or predictable prognosis; and conditions with uncertain prognosis, in which decision making is guided by the best interests of the fetus/newborn [7]. This approach reinforces the heterogeneity of these conditions and supports a more structured understanding to guide perinatal palliative care planning.

Most articles found in the present review address termination of pregnancy in cases of life-limiting conditions, with palliative care protocols being recommended for families who choose to maintain the pregnancy [6,7,9,10,11,16,17,18,19,20,21,22,23,24]. Two of the four Brazilian studies bring the national reality on the subject, pointing out that to terminate the pregnancy in these cases, besides anencephaly (the only malformation provided for by law for termination of pregnancy), judicial authorization is required [3,7]. The decisions vary according to cultural, social, and religious issues and are directly influenced by the laws regarding abortion.

The implementation of perinatal palliative care is profoundly shaped by local legal, religious, cultural, and resource-related contexts, which must be considered when interpreting and adapting the evidence. In the United States, where most included studies originated, access to perinatal palliative care programs is expanding, supported by professional society guidelines such as those from ACOG. In contrast, in Brazil, pregnancy termination is legally restricted to cases of anencephaly, sexual violence and risk to the mother’s life, which means that families facing other life-limiting fetal diagnoses must continue the pregnancy, making palliative care pathways not only appropriate but ethically necessary. In several European countries such as Italy, Spain, and Poland, religious values and legal restrictions similarly influence decision making, although with varying degrees of institutional integration of palliative care. In Poland, where termination of pregnancy has been further restricted by recent legislation, the importance of structured perinatal palliative care pathways has grown considerably. These contextual differences reinforce that no single protocol is universally applied.

In the care of families who continue the pregnancy in the context of a limiting condition, it is important to understand that some will remain in denial or will not accept the fetal diagnosis. However, if an intervention ceases to contribute to the patient’s quality of life, it is recommended to reevaluate the treatment. The suspension or non-introduction of invasive measures may be ethically appropriate in situations of guarded prognosis or life-limiting condition [3,6,12,13]. In the face of possible divergences between professionals or between the health team and the family, new conversations, offering a second opinion, or performing additional complementary exams, can help in decision making [9,10,16].

In this perspective, when planning the type of neonatal care in the context of limiting conditions, there is a difficulty on the part of health professionals in indicating palliative care during the gestational period. Even with all the diagnostic tools, there are still cases of uncertain prognosis or those where the neonatal diagnosis may be different from the antenatal one. The team needs to be prepared to work with uncertainties, as well as to include the pregnant woman and family members, starting from prenatal care, in the discussion about the existing possibilities [9,10,15,19,20,23]. This can support the idea that perinatal decision making should also consider evidence-based obstetric and neonatal interventions which may reduce long-term neurological morbidity in selected groups, but always in line with prognosis, goals of care and family preferences [27].

In addition to the uncertainties addressed in family meetings, it is important to reduce divergences among team members. For this, it is possible to hold case discussion meetings to plan the conduct [3,10,13,17], to review the attitudes taken in resolved cases [4,16]. And also, to promote mutual support among professionals [6,11,12,16,20]. Continuing education for professionals and training in palliative care are essential elements to provide adequate assistance.

By gathering these different protocols and experiences, this study seeks not only to present an overview of the existing approaches, but also to contribute to the development of future research. Despite the growing recognition of perinatal palliative care, its implementation across different countries and healthcare settings remains difficult. Although protocols exist in some institutions, their integration into prenatal care is often limited by the absence of structured referral pathways. Barriers also include insufficient professional training, lack of continuing education and absence of standardized approaches. Uncertainty in fetal prognosis further complicates decision making and may delay referral, especially when combined with variable perceptions among healthcare providers about the role of palliative care.

Beyond the descriptive aggregation of studies, it is possible to identify recurring structural components across different models of perinatal palliative care. These components can be grouped into core domains: (1) early and structured communication at diagnosis; (2) multidisciplinary and longitudinal prenatal follow-up; (3) shared decision making and individualized birth planning; (4) alignment of intrapartum and neonatal care with goals of care; and (5) structured bereavement support. These domains are not independent but interrelated, forming a continuum in which each component reinforces the others.

This study aimed to provide an integrated synthesis of existing protocols and care models, with a specific focus on prenatal care. This review organizes the available evidence into a structured continuum of care, from diagnosis to bereavement. Additionally, it highlights common elements across different healthcare systems and proposes a practical framework that may support the development or adaptation of protocols, particularly in settings where formal programs are not yet established.

Future protocols and care models in perinatal palliative care should incorporate standardized outcome measures to enable evaluation and comparison across settings. Relevant domains include parental satisfaction with communication and decision making, decisional regret, birth plan completion rates, neonatal comfort as assessed by validated scales, quality of bereavement follow-up, and psychological outcomes in the postpartum period, including rates of complicated grief and post-traumatic stress. The development of consensus-based quality indicators specific to perinatal palliative care represents a priority area for future research.

This review has several limitations that should be acknowledged. The search strategy was intentionally broad but simplified, aiming to capture studies addressing both clinical practice and conceptual models in a heterogeneous and evolving field. However, the use of a limited number of descriptors and databases may have reduced sensitivity and failed to identify all relevant studies indexed under alternative terms. A more comprehensive strategy using MeSH terms, additional databases, and complex Boolean operators could improve reproducibility and coverage in future reviews. The proposed integrated model should be interpreted as a conceptual synthesis derived from recurring elements identified in the literature, rather than as a validated or universally applicable framework. Its purpose is to provide an organizational structure to support clinical reasoning and future research, not to prescribe a standardized protocol. Studies published in English, Portuguese, and Spanish were included due to feasibility and the authors’ ability to accurately interpret the data. Although this approach allowed inclusion of both the international and regional literature, it may have introduced language bias by excluding studies published in other languages. Study selection was conducted by a single reviewer, which may increase the risk of selection bias and affect the completeness and balance of the included evidence. Therefore, the findings should be interpreted as a structured but non-exhaustive synthesis rather than a comprehensive or definitive representation of all available evidence. As a narrative review, this study did not follow a systematic approach to study selection and appraisal. The included studies are methodologically heterogeneous, comprising narrative reviews, expert recommendations, qualitative studies (interviews and surveys), retrospective chart reviews, and descriptions of institutional care programs. This diversity reflects the current developmental stage of perinatal palliative care as a clinical field but limits direct comparability between studies. Additionally, no formal critical appraisal or grading of evidence was performed. This decision reflects both the narrative design and the marked heterogeneity of the included literature, which comprises qualitative studies, expert recommendations, institutional protocols, and reviews. Accordingly, the aim was not to establish a hierarchy of evidence, but to identify recurring conceptual elements across different sources. These methodological choices may limit the ability to assess the relative strength of the evidence; however, they are consistent with the exploratory and integrative purpose of this review, which is to provide a structured conceptual framework and support the development of future research in this field.

## 5. Conclusions

There are many common points in the articles found, such as the encouragement of effective and compassionate communication, family-centered care, prenatal planning and birth plan, neonatal comfort measures, grief preparation, and the participation of a multidisciplinary team, which are essential for promoting comprehensive perinatal palliative care. The application of this approach varies according to social issues, available resources, legislation, and culture, making it necessary to adapt the care according to the local context. Ultimately, perinatal palliative care is not limited to end of life care, but represents an approach centered on quality of life, symptom management, and comprehensive support in the context of life-limiting or life-threatening conditions. The present review further highlights that perinatal palliative care is, in its essence, a personalized medicine practice: its effectiveness depends on precise diagnosis, individualized prognostic counseling, patient and family-centered decision making, and flexible adaptation of care protocols to the specific characteristics of each case.

Future studies should focus on validating a minimal set of protocol components through consensus-based methods, defining measurable quality indicators, such as referral time, birth plan completion rates, parental satisfaction, alignment of care with parental preferences, and psychological outcomes after perinatal loss, to support the transition from experience-based models to reproducible, scalable interventions.

## Figures and Tables

**Table 1:** Selected articles on perinatal palliative care.

Authors	Year/Country	Study	Key Contribution
Carter [8]	2017, USA	Expert opinion	Safety for families and professionals; Does not alter outcomes; Counseling more aligned with cultural values.
Côté-Arsenault and Denney-Koelsch [9]	2016, USA	Qualitative interview study	Stages: pre-diagnosis, learning the diagnosis, living with the diagnosis, birth and death, and post-death; Tasks: navigating relationships, understanding the implication of the condition, reviewing pregnancy goals, promoting more time with the baby, preparing for birth and death, advocating for the baby, adjusting to life without the baby; The use of a flowchart helps health professionals, improving family satisfaction.
Sidgwick et al. [10]	2016, UK	Model of care (Expert opinion)	Practical model for consultations: establishing diagnosis, discussing palliative care (PC) with the family, birth plan, and place of care; Clinical case to illustrate the approach taken; Emphasizes shared decision making and planning with a multidisciplinary team; Presents a list of conditions and candidates for PC; Personalized prenatal, delivery, and postpartum plans.
Catania et al. [7]	2017, Brazil	Systematic review	Recommendations for adopting PC, from the moment of decision, prenatal care, delivery, transition to death, and post-death; Lists eligible conditions; Training of healthcare professionals.
Cole et al. [11]	2017, USA	Care program	Program steps: diagnosis, prenatal consultation, birth plan, and family support; Clinical case to illustrate the approach taken; Multidisciplinary approach to the birth plan; Patient and family welcomed from a “mind, body, and spirit” perspective.
Bolibio et al. [3]	2018, Brazil	Care program	Model of PC applied to pregnant women and families; Describes candidates, prognosis evaluation, follow-up during prenatal care, birth plan, delivery, and postpartum.
Wool and Catlin [12]	2019, USA	Care program	Integration of grief care; Highlights rituals and memories; Indicates professional training failures; Trained professionals.
Rusalen et al. [13]	2019, USA	Care program (review)	Palliative care divided into three groups: extreme prematurity, list of life-limiting conditions, neonates in high complexity intensive care. Recommends prenatal follow-up, psychological support, and birth plan (vaginal recommended); Comfort measures for the neonate.
Cortezzo et al. [14]	2019, USA	Interview (physicians and parents)	Interviews with patients and professionals; Families emphasized the need for creating memories and reported that the birth plan brings a sense of control; Physicians report tranquility with the birth plan, with barriers such as time and training; Communication between families and neonatal team to align expectations; Lists fetal conditions of research participants.
American College of Obstetricians and Gynecologists (ACOG) [6]	2019, USA	Protocol	Recommendations for family care; Patient selection and components of care, such as diagnosis, follow-up, birth plan, access to pediatric specialties, care during delivery and postpartum, and grief support; Health services should develop PC programs.
Falke and Baas [4]	2020, USA	Care program	Program receives palliative care referrals, but qualification is lacking; Development of guidelines can improve the uniformity of referrals; Continuous evaluation of the protocol; Professional training; Involving families in care.
Linebarger [15]	2020, USA	Expert opinion	Emotionally support parents facing uncertainty, ensuring they feel respected and that the life matters; Different forms of reaction and attitudes from families.
Lago et al. [16]	2020, Italy	Literature review	Defines indications and candidates; Generates quality indicators for the implemented protocols; Staff education; A palliative care program is mandatory to guide physicians and parents.
Bernardes et al. [17]	2020, Brazil	Family conference study	Describes the themes from family meetings; Care flowchart, “CEPOS”: talk about the illness, understand the context of the moment, prepare the care, organize the moment of delivery and follow-up (postpartum); List of diagnoses of the participants. PC is very important, especially in countries that do not permit termination of pregnancy.
Ziegler et al. [18]	2020, USA	Care program	Care model, including location, candidates (life-limiting conditions listed), family and local health service support, and grief care; Recommendations for implementation in other hospitals.
Cortezzo et al. [19]	2020, USA	Care program	Wide list of candidates; Role of the birth plan: families can express their fears, values, hopes, and desires; health professionals have the opportunity to access these wishes during pregnancy, delivery, birth, and postpartum; The grief process begins even before the family meets the baby; Multidisciplinary team; Reduces maternal stress and promotes family-centered care.
Martín-Ancel et al. [20]	2022, Spain	Care program	Care model structured with a list of diagnoses; Comfort measures for the neonate; Prenatal care with a multidisciplinary team; Grief support, post-mortem care, and breastfeeding guidance; Training for professionals.
Tataj-Puzyna et al. [21]	2023, Poland	Family conference study	Paths chosen by families who had a diagnosis of a life-limiting condition: “Search for normality”, “Search for communitas”, and “Search for an individual path”; There is no single method of prenatal education for families; Promotion of meetings among families, but with caution, as the varied diagnoses lead to distinct experiences; Fetal diagnoses were listed; The birth plan allows alignment of parental expectations; Parents should have the option of various paths for preparing for birth, which best meet their preferences.
Alves et al. [22]	2023, Brazil	Literature review	Strategies for referral to PC, such as prioritized psycho-spiritual support for the family, and care options to evaluate the limits of invasive care; Training of professionals and organization of services; Lack of professional preparation to approach the families.
Henry and Cote-Arsenault [23]	2024, USA	Case study	Clinical case to illustrate the approach taken; Interprofessional and family-centered care; Previously described model, with five stages of pregnancy: pre-diagnosis, learning the diagnosis, living with the diagnosis, birth and death, and post-death; Continuing education.
Korzeniewska-Eksterowicz et al. [24]	2025, Poland	Review of medical records	Out of 72 pregnant women, 68 decided to continue the pregnancy and received PC; Majority of newborns died in the first week of life; Grief assistance, ensuring memory creation; List of diagnoses; Improving the referral process.

**Table 2:** Concepts of fetal or neonatal life-limiting condition (LLC).

Author, Year	Concept
Côté-Arsenault and Denney-Koelsch, 2016 [9]	LLC (Life-Limiting Condition) implies that the malformation will shorten the baby’s life, while “lethal fetal diagnosis” is defined as a condition that leads to death at any point up to two to three months after birth.
Sidgwick et al., 2016 [10]	LLCs have specific diagnosis and prognosis, including a very high chance of death in utero or in childhood, despite medical treatment.
Bolibio et al., 2018 [3]	LLC includes most major fetal malformations (malformations that require surgery or that lead to restriction in the length or quality of life).
ACOG 2019 [6]	LLC includes lethal fetal conditions, as well as others for which there is little or no prospect of long-term extrauterine survival without severe morbidity or extremely low quality of life and for which there is no cure.
Lago et al., 2020 [16]	LLCs or life-threatening conditions appear incompatible with long-term survival and/or carry the risk of severely compromising the quality of life (adapted).
Ziegler et al., 2020 [18]	LLC presents a life expectancy of only months, not years.
Cortezzo, 2020 [19]	LLC is a condition that has a low probability of long-term survival without severe morbidity that may affect the quality of life.
Falke and Baas, 2020 [4]	LLC: there is no reasonable hope or cure, and the children will eventually die prematurely.
Martín-Ancel et al., 2022 [20]	LLCs (with no chance of cure) are diseases that lead to death in utero or in the first hours or days after birth; Progressive disease that will cause death in a few months or years; Non-progressive and irreversible diseases that cause severe disability and are associated with a higher risk of health complications.
Tataj-Puzyna et al., 2023 [21]	LLCs are a confirmed state or condition of the fetus or baby that may lead to death before or after birth.

## Data Availability

No new data were created or analyzed in this study. Data sharing is not applicable to this article.
